# Osteoma cutis masquerading as an ingrowing toenail: a case report

**DOI:** 10.4076/1757-1626-2-7176

**Published:** 2009-07-16

**Authors:** Andrew G Titchener, Darryl N Ramoutar, Hussein Al-Rufaie, Daniel T Rajan

**Affiliations:** 1 Department of Trauma and Orthopaedic Surgery, Hinchingbrooke HospitalHuntingdon, PE29 6NTUK; 2 Department of Pathology, Hinchingbrooke HospitalHuntingdon, PE29 6NTUK

## Abstract

Osteoma cutis of the foot is extremely rare and there are very few reported cases. The incidence of in-growing toenail in the United Kingdom is estimated to be 10,000 new cases per year and many are treated non-operatively. We present a case where osteoma cutis was masquerading as an in-growing toenail, and wish to highlight the condition as a differential diagnosis for this condition. There have been case reports of bony cutaneous lesions of the foot, both benign and malignant and so these are especially important to consider in the differential diagnoses where non-operative management is being considered.

## Introduction

Osteoma cutis is an excessively rare lesion which may be either primary or secondary to neoplastic or inflammatory conditions [1]. Primary lesions are defined as such in the absence of a preceding skin lesion. Secondary lesions are more common and are associated with scar tissue, acne vulgaris, melanocytic naevi, and basal cell carcinoma. Osteoma cutis may occur at any age and in either sex and has been reported on the hands [2], but there are few reported cases of foot lesions [3]. It has been found to simulate verruca plantaris [4] as well as heel pain [3], but lesions are far more frequently reported on the head and neck of white female patients. There are a number of syndromes associated with osteoma cutis, such as Albright’s osteodystrophy [5], fibrodysplasia ossificans and progressive osseous heteroplasia. The stimulus for osteoma formation is unknown.

## Case presentation

A 30-year-old otherwise fit and healthy white British male was referred to our clinic by his general practitioner with an ingrowing toenail of his right hallux from which he had suffered since childhood. Examination of the foot revealed no evidence of infection or cellulitis. The hallux nail was in-growing on both its edges, and there was firm granulation tissue palpable at the lateral nail fold. The adjacent interphalangeal joint was normal to examination. A radiograph of the foot revealed no evidence of osteomyelitis.

**Figure 1.**
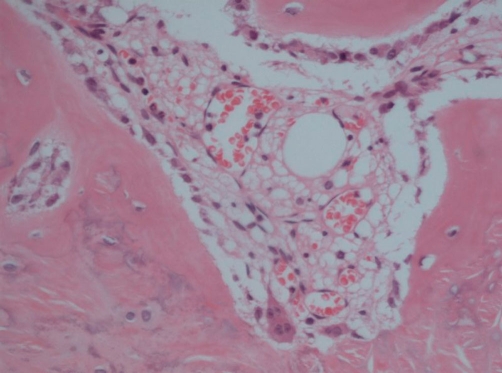
Mature lamellar bone containing marrow spaces, representing osteoma cutis.

The patient underwent a total excision of the toe nail. At operation a small bony cutaneous lump underlying the nail bed was excised in toto and sent for histopathological examination. This revealed a well circumscribed dermal nodule of mature lamellar bone containing marrow spaces, which represents osteoma cutis (Figure 1). At four week follow up the nail bed was healing well and there was no evidence of any residual cutaneous lesions.

## Discussion

The incidence of in-growing toenail in the UK is estimated to be 10,000 new cases per year [6]. In patients in whom it causes symptoms, it is often excised and the nail bed cleared, however non operative management of in-growing toenail is an accepted modality of management. When the nail punctures the skin, granulation tissue is produced at the nail margin. What appears to be a primary ingrowing toe nail could sometimes be secondary to other causes such as soft tissue chondroma [7] osteochondroma [8], extraskeletal osteosarcoma [9] as well as osteoma cutis; the latter which is rare is highlighted by this case report.

## References

[bib-001] Conlin PA, Jimenez-Quintero LP, Rapini RP (2002). Osteomas of the Skin Revisited: A Clinicopathologic Review of 74 Cases. Am J Dermatopathol.

[bib-002] Boschert MT, Puckett CL (2000). Osteoma cutis of the hand. Plast Reconstr Surg.

[bib-003] Klein MD (1995). Primary osteoma cutis. J Am Podiatr Med Assoc.

[bib-004] Thompson RG (1956). Primary osteoma cutis; report of a case simulating verruca plantaris. AMA Arch Derm.

[bib-005] Cortes W, Gosain AK (2006). Recurrent ectopic calcification involving the maxillofacial skeleton: A potential harbinger of Albright’s osteodystrophy. J Craniofac Surg.

[bib-006] Sykes PA (1986). Ingrowing toenails: Time for critical appraisal?. J R Coll Surg Edinb.

[bib-007] Anthouli-Anagnostopoulou FA, Papachristou G (2000). Extraskeletal chondroma, a rare soft tissue tumor. Case report. Acta Orthop Belg.

[bib-008] Sheff JS, Wang S (2005). Extraskeletal osteochondroma of the foot. J Foot Ankle Surg.

[bib-009] Hulse N, Paul AS (2006). Soft tissue Osteosarcoma: A Case Report. Acta Orthop. Belg.

